# Supplementary material for: Risk of Malnutrition in Hospitalized COVID-19 Patients: A Systematic Review and Meta-Analysis
Source: Nutrients. 2022 Dec 10;14(24):5267. doi: 10.3390/nu14245267 (PMC9780808; doi:10.3390/nu14245267)
Supplement: Supplementary file 1 [file nutrients-14-05267-s001.zip › nutrients-2012469-supplementary.pdf]

# Risk of Malnutrition in Hospitalized COVID-19 Patients - a Systematic Review and Meta-analysis
## Supplementary Material

**Review protocol registration**

PROSPERO 2022 CRD42022338383 Available from: https://www.crd.york.ac.uk/prospero/display_record.php?ID=CRD42022338383

**Table S1. Search Strategy.**

| Source | Strategy |
|---|---|
| **PubMed (N=1299)** | (Covid-19 [Title/Abstract] OR SARS-CoV-2[Title/Abstract] OR 2019 Novel Coronavirus Disease [Title/Abstract]) AND ((malnutrition [Title/Abstract] OR malnutrition [MeSH Terms] OR nutritional deficiency [Title/Abstract] OR nutritional status [Title/Abstract]) OR (sarcopenia [Title/Abstract] OR sarcopenia [MeSH Terms] OR muscle Loss [Title/Abstract] OR muscular atrophy [MeSH Terms]) OR (cachexia [MeSH Terms])) AND ("2019/12/1"[Date - Publication] : "2022/8/31"[Date - Publication]) |
| **Web of Science (N=1432)** | (AB=(Covid-19 OR SARS-CoV-2 OR 2019 Novel Coronavirus Disease) OR TI=(Covid-19 OR SARS-CoV-2 OR 2019 Novel Coronavirus Disease)) AND ((AB=(malnutrition OR malnutrition OR nutritional deficiency OR nutritional status) OR TI=(malnutrition OR malnutrition OR nutritional deficiency OR nutritional status)) OR (AB=(sarcopenia OR sarcopenia OR muscle Loss OR muscular atrophy) OR TI=(sarcopenia OR sarcopenia OR muscle Loss OR muscular atrophy)) OR (AB=(cachexia) OR TI=(cachexia))) AND DOP=(2019-12-01/2022-08-31) |
| **EMBASE (N=1698)** | ('Covid-19':ab,ti OR 'SARS-CoV-2':ab,ti OR '2019 Novel Coronavirus Disease':ab,ti) AND (('malnutrition':ab,ti OR 'malnutrition'/exp OR 'nutritional deficiency':ab,ti OR 'nutritional status':ab,ti ) OR ('sarcopenia':ab,ti OR 'sarcopenia'/exp OR 'muscle Loss':ab,ti OR 'muscular atrophy'/exp) OR ('cachexia'/exp)) AND [embase]/lim AND [01-12-2019]/sd NOT [31-08-2022]/sd |

**Table S2. Study characteristics by other malnutrition or risk of malnutrition assessment tools**

| Author | Publication year | Country | Time frame | Populaiton size | Age [1] | Sex | Race/ethnicity | COVID-19 confirmation | Severity of COVID-19 | Outcome | Assessment tool and definition | Prevalence | Unified prevalence [2] |
|---|---|---|---|---|---|---|---|---|---|---|---|---|---|
| **General ward - at admission** | | | | | | | | | | | | | |
| Da et al. [1] | 2021 | Italy | 2020/1/1-2020/2/28 | 150 | 69 (58–78) | Male (68.7 %) | NR | RT-PCR/CT | Clinical severity of COVID-19 pneumonia (WHO stage):13.3% stage0;33.3% stage1;38.6% stage2;14.8% stage3 | Malnutrition (Y/N) | BIVA;malnutrition = when the tip of the impedance vector was within the lower right quadrant outside the 75th percentile of the tolerance ellipse along the horizontal axis | 24.70% | 24.7% |
| Wierdsma et al. [2] | 2021 | Netherlands | 2020/4/27-2020/12/31 | 162 | NR | NR | NR | NR | NA | Malnutrition (Y/N) | BMI;malnutrition = BMI <18.5 kg/m2 or when recent weight loss was present: >5% in 1 week and/or >10% in 1 month and/or >12% lower than their regular body weight | 36% | 36.0% |
| Zhou et al. [3] | 2021 | China | 2019/12-2020/3 | 429 | 58.29 ± 15.89 | Male (49.42 %) | NR | NR | NA | Malnutrition risk (low/high) | CONUT; 0–4=the low risk, 5–12= high risk | 34.3% low risk;65.7% high risk | 65.7% |

| | | | | | | | | | | | | | |
|---|---|---|---|---|---|---|---|---|---|---|---|---|---|
| Song et al. [4] | 2021 | China | 2020/1-2020/5 | 295 | 58.00 (44.00–69.00) | Male (52.54 %) | NR | CT | 16.9%mild; 62.0% moderate; 22.4% severe; 13.9% critical | Malnutrition(light/moderate/severe) | CONUT;0-1=no malnutrition, 2–4 =light malnutrition, 5–8 =moderate malnutrition, 9–12 = severe malnutrition | 33.04% no malnutrition;36.61% light Malnutrition; 20.54% moderate malnutrition; 9.82% severe Malnutrition | 30.4% |
| You et al. [5] | 2022 | China | 2020/2/9-2020/3/31 | 91 | 60.5±15.9 | Male (57.1 %) | NR | PCR/CT | 100% severe or critical | Malnutrition (Y/N) | GLIM | 18.90% | 18.9% |
| Recinella et al. [6] | 2020 | Italy | 2020/3/30-2020/5/15 | 109 | 83 (76-91.5) | Male (51.4 %) | NR | RT-PCR | NA | Malnutrition risk(no/low/severe-moderate) | GNRI; > 98=no risk, 92–98=low risk, < 92=severe–moderate risk | 27.5% no risk;11% low risk; 61.5% moderate-severe risk | 61.5% |
| Otero et al. [7] | 2021 | Spain | 2020/4 | 83 | 82 (75–88) | Male (42.2 %) | NR | PCR | NA | Malnutrition(no/risk/yes) | MNA; ⩾12 = no malnutrition, 8–11 = at risk of malnutrition, ≤7 = malnutrition | 27.7%no malnutrition;37.3% at risk of malnutrition ;34.9% malnutrition | 72.2% |
| McGovern et al. [8] | 2022 | United Kingdom | 2020/4/1-2020/7/6 | 106 | < 70,33.0%;≥ 70,67.0% | Male (52.8 %) | NR | RT-PCR/CT | NA | Malnutrition risk (Y/N) | MUST;0 = no risk, ≥1 = at risk | 42.40% | 42.4% |
| Larrazabal et al. [9] | 2021 | Philippines | 2020/7/15-2020/9/15 | 355 | NR | NR | NR | RT-PCR | NA | Malnutrition risk(low/moderate/high) | NRS-2002; 0–2 = low risk, 3–5 = moderate risk, ≥ 6 = high risk | 37.7% low risk; 47.3% moderate risk; 14.9% high risk | 62.2% |
| Mendes et al. [10] | 2021 | Switzerland | 2020/3/13-2020/5/17 | 245 | 86.1 ± 6.4 | Male (42%) | NR | RT-PCR | NA | Malnutrition risk(no/risk/high) | NRS-2002;<3= no malnutrition risk, ≥3 = at risk of malnutrition, ≥ 5 high malnutrition risk | 17.6% no malnutrition; 32.2% at risk of malnutrition; 50.2% high malnutrition | 82.4% |

| | | | | | | | | | | | | | |
|---|---|---|---|---|---|---|---|---|---|---|---|---|---|
| Del Giorno et al. [11] | 2020 | Switzerland | 2020/3 | 90 | 64.5±13.7 | Male (67.8 %) | NR | PCR/CT | NA | Malnutrition risk (Y/N) | NRS-2002; ≥3 = at risk of malnutrition | 92.0% | 92.0% |
| Martin–Martinez et al. [12] | 2021 | Spain | 2020/4/14-2020/7/30 | 205 | 69.3 ± 17.5 | Male (47.8 %) | NR | PCR | NA | Malnutrition risk (Y/N) | NRS-2002; ≥3 = at risk of malnutrition | 88.7% | 88.7% |
| Voelkle et al. [13] | 2022 | Switzerland | 2020/3/17-2020/4/30 | 57 | 67.0 (60.0, 74.2) | Male (60%) | NR | RT-PCR | NA | Malnutrition risk (Y/N) | NRS-2002; ≥3 = at risk of malnutrition | 19.0% | 19.0% |
| Youssef et al. [14] | 2022 | Egypt | 2020/7-2020/12 | 121 | 52.37±10.48 | Male (84.3 %) | NR | NR | NA | Malnutrition risk (Mild–moderate/Severe) | NRS-2002;1–2=Mild–moderate risk of malnutrition, ≥3=Severe risk of malnutrition | 94.9%Mild–moderate risk; 5.1% Severe risk | 5.1% |
| Zhang et al.[15] | 2021 | China | 2020/2/6-2020/3/20 | 101 | 65.3 ± 13 | Male (59.4 %) | NR | NR | 53.4% severe | Malnutrition(no/risk/yes) | NRS-2002+SGA;NRS-2002 <3 = no risk for malnutrition, NRS-2002 ≥ 3 with SGA A= risk for malnutrition, NRS-2002 score ≥ 3 and SGA B/C = malnourished | 59.4% no risk for malnutrition; 22.7% risk for malnutrition; 17.8% malnutrition | 40.5% |
| Zhao et al. [16] | 2021 | China | 2020/1/29-2020/2/19 | 371 | NR | NR | NR | NR | 83.6%severe; 16.4% critical | Malnutrition risk (no/low/high) | NRS-2002;<3= no malnutrition risk, ≥3 = at risk of malnutrition, ≥ 5 high malnutrition risk | 8% no risk;76% low risk;16% high risk | 92.0% |

| | | | | | | | | | | | | | |
|---|---|---|---|---|---|---|---|---|---|---|---|---|---|
| Cui et al. [17] | 2021 | China | 2020/1/29-2020/2/19 | 408 | 61.0±12.6 | Male (51.5 %) | NR | PCR | 82.9% severe;17.1% Critical | Malnutrition risk(Y/N) | NRS-2002; ⩾4 = at risk of malnutrition | 48% | 48% |
| Fernandes et al. [18] | 2022 | Brazil | 2020/6/2-2020/7/21,Clinical Hospital of the School of Medicine of the University of São Paulo;2020/7/22-2020/9/25Ibirapuera Field Hospital | 309 | 55.8±13.9 | Male (53.4 %) | 48.9% White; 36.6% Pardo;14.2 % Black; 0.3% Asian | RT-PCR/CT | NA | Malnutrition (no/mild/yes) | PNI;<40=malnutrition, 40–45=mild malnutrition, > 45=non-malnutrition | 9.4% non-malnutrition; 19.1% mild malnutrition; 71.5% malnutrition | 71.5% |
| Liu et al. [19] | 2021 | China | 2020/2/14-2020/3/14 | 47 | NR | Male (48.9 %) | NR | PCR | NA | Malnutrition(no/risk/yes) | SGA (no malnutrition, at risk of malnutrition,malnutrition) | 44.7%% no malnutrition;38.3% at risk of malnutrition ;17.0% malnutrition | 55.3% |
| **General ward - during hospitalization** | | | | | | | | | | | | | |
| Allard et al. [20] | 2020 | France | 2020/4/9-2020/5/29 | 108 | 61.8 ± 15.8 | Male (59.3 %) | 32.7% Caucasian;32.7% Arabic; 18.7% Afro-Caribbean; 15.9% Asian | PCR/CT | 68.5% Non-severe;31.5% Severe | Malnutrition (Y/N) | BMI; malnutrition = if at least one of the three following criteria was present: body mass index (BMI) < 18.5 kg/m2 (or < 21.0 kg/m2 if age was ≥ 70 years), weight loss ≥ 5% in the previous | 38.9% | 38.9% |

| | | | | | | | | | | | | | |
|---|---|---|---|---|---|---|---|---|---|---|---|---|---|
| | | | | | | | | | | | month, and/or ≥ 10% in the previous six months | | |
| Wei et al. [21] | 2020 | China | 2020/1/3-2020/3/11 | 348 | 66.0 (56.0–73.0) | Male (52.3 %) | NR | NR | 100% severe | Malnutrition (no/mild/severe) | CONUT;0–1 = no malnutrition, 2–4=mild malnutrition, ≥5=moderate-severe malnutrition | 13.8% no malnutrition;46.3% mild malnutrition ;39.9% severe malnutrition | 39.9% |
| Yu et al. [22] | 2020 | China | 2020/2-2020/4 | 139 | ≥65 years(41.70%) | Male (51.8 %) | NR | RT-PCR/CT | NA | Malnutrition (Y/N) | GLIM | 17.98%(6.57% moderate; 9.35% severe) | 18.0% |
| Rouget et al. [23] | 2021 | France | 2020/3-2020/4 | 80 | 59·5(Median) | Male (75%) | NR | RT-PCR | NA | Malnutrition (Y/N) | GLIM | 37.5% (26.25% severe malnutrition) | 37.5% |
| Bedock et al. [24] | 2020 | France | 2020/3/21-2020/4/24 | 114 | 59.9 ± 15.9 | Male (60.5 %) | NR | RT-PCR/CT | NA | Malnutrition (no/moderate/severe) | GLIM | 57.9% no malnutrition;23.7% moderate malnutrition;18.4% severe malnutrition | 42.1% |
| Martin–Martinez et al. [12] | 2021 | Spain | 2020/4/14-2020/7/31 | 205 | 69.3 ± 17.6 | Male (47.9 %) | NR | PCR | NA | Malnutrition (Y/N) | GLIM | 45.50% | 45.5% |
| Vaillant, et al. [25] | 2022 | France | 2020/5/7-2020/7/11 | 403 | 62.2 ± 14.3 | Male (64%) | NR | NR | NA | Malnutrition (Y/N) | GLIM | 67%(42% severe) | 67.0% |

| | | | | | | | | | | | | | |
|---|---|---|---|---|---|---|---|---|---|---|---|---|---|
| Li et al. [26] | 2020 | China | 2020/1-2020/2 | 182 | 68.5±8.8 | Male (35.7 %) | NR | PCR | NA | Malnutrition(no/risk/yes) | MNA;<17 = malnutrition, 17–23.5 = risk of malnutrition; 24–30 =non-malnutrition | 19.8% no malnutrition;27.5% at risk of malnutrition; 52.7% malnutrition | 80.2% |
| Thiam et al. [27] | 2022 | Malaysia | 2021/3/1-2021/5/31 | 21 | NR | NR | NR | NR | NA | Malnutrition risk(no/risk/yes) | MNA-SF; 0–7=malnutrition, 8–11=at risk of malnutrition, 12–14= no malnutrition | 61.1% no malnutrition ;18.5% at risk of malnutrition;17.9% malnutrition | 36.4% |
| Farina et al. [28] | 2021 | US | 20203/1-20205/1 | 155 | 60.3±13.8 | Male (66.5 %) | NR | PCR | NA | Malnutrition risk (Y/N) | mNUTRIC; ≥ 5=at risk of malnutrition | 72.90% | 72.9% |
| Fiorindi et al. [29] | 2021 | Italy | 2020/3-2020/4 | 142 | 60(Mode);61(Median) | Male (58.5 %) | NR | PCR | NA | Malnutrition (Y/N) | MUST | 8.40% | 8.4% |
| Stefano et al. [30] | 2021 | Italy | 2020/1/3-2020/4/30 | 515 | NR | Male (68.5 %) | | | NA | Malnutrition risk(no/average/higher) | MUST; 1=average risk of malnutrition, ≥2=higher risk of malnutrition | 82% no malnutrition risk;2.5% average risk of malnutrition ;15.5% higher risk of malnutrition | 18.0% |
| Vong et al. [31] | 2022 | US | 2020/3/1-2020/12/3 | 4311 | NR | Male (49.2 %) | 4.6% Asian;36.5 % Black; 36.1% White; 22.8% Other | PCR | 55.1% mild or moderate; 10% severe | Malnutrition risk (Y/N) | MUST | 9.30% | 9.3% |
| Ansu et al. [32] | 2021 | US | 2020/5/19-2020/7/2 | 77 | 63.48 ± 14.98 | NR | NR | NR | NA | Malnutrition (Y/N) | NCPT | 18.40% | 18.4% |

| | | | | | | | | | | | | |
|---|---|---|---|---|---|---|---|---|---|---|---|---|
| Pimentel et al. [33] | 2021 | Brazil | NR | 41 | 45.7±12.4 | Male (61.0 %) | NR | RT-PCR | NA | Malnutrition risk (low/high) | NRS-2002;<3 = low malnutrition risk,≥3 = high malnutrition risk | 22.0% low malnutrition risk; 78.0% high malnutrition risk | 78.0% |
| Pironi et al. [34] | 2021 | Italy | 2020/4 | 162 | NR | NR | NR | NR | NA | Malnutrition risk(Y/N) | NRS-2002;<3 = no malnutrition risk,≥3 = at risk of malnutrition | 67.3% | 67.3% |
| Shabanpur et al. [35] | 2022 | Iran | 2021/5-2021/7 | 330 | NR | NR | NR | NR | NA | Malnutrition (risk/moderate/severe) | NRS-2002;<3=at risk, 3–4 = moderate malnutrition, ≥ 5 = severe malnutrition | 4% at risk; 69%moderate malnutrition; 27% severe malnutrition | 96.1% |
| Liu et al. [36] | 2020 | China | 2020/1/28-2020/3/28 | 141 | 71.688±5.851 | Male (48.2 %) | NR | RT-PCR/CT | NA | Malnutrition risk (Y/N) | NRS-2002; ⩾3 = at risk of malnutrition | 85.1% | 85.1% |
| Liu et al. [37] | 2021 | China | 2020/1/29-2020/3/15 | 760 | 60 (46, 74) | Male (50%) | NR | RT-PCR/CT | NA | Malnutrition risk (Y/N) | NRS-2002; ⩾3 = at risk of malnutrition | 82.6% | 82.6% |
| Nicolau et al. [38] | 2021 | Spain | 2020/4-2020/6 | 75 | NR | Male (49.5 %) | NR | PCR | NA | Malnutrition (Y/N) | SGA; Grade A =no malnutrition, Grade B= mild/moderate malnutrition,Grade C =severe malnutrition;Malnutrition= SGA score of B (mild/moderate) or C (severe) | 36% (22.7%mild/moderate;13.3% severe) | 36.0% |
| **ICU - at admission** | | | | | | | | | | | | | |

| | | | | | | | | | | | | | |
|---|---|---|---|---|---|---|---|---|---|---|---|---|---|
| Wierdsma et al. [2] | 2021 | Netherlands | 2020/4/27-2020/12/31 | 245 | NR | NR | NR | NR | NA | Malnutrition (Y/N) | BMI;malnutrition = BMI <18.5 kg/m2 or when recent weight loss was present: >5% in 1 week and/or >10% in 1 month and/or >11% lower than their regular body weight | 34% | 34.0% |
| Rives-Lange et al. [39] | 2021 | France | 2020/3/15-2020/5/15 | 38 | 66 (59-72) | Male (76%) | NR | PCR | NA | Malnutrition (no/moderate/severe) | BMI;Moderate malnutrition= weight loss between 5 and 10% within the past 6 months or 10–20% beyond 6 months and/or BMI <20 kg/m2 (if age <70 years) or BMI <22 kg/m2 (if age ≥70). Severe malnutrition = weight loss over 10% within the past 6 months or over 20% beyond 6 months and/or BMI <18.5 kg/m2 (if age <70 years) or BMI <20 kg/m2 (if age ≥70) | 82% no malnutrition;13% moderate malnutrition ;5% severe malnutrition | 18.0% |

| | | | | | | | | | | | | | |
|---|---|---|---|---|---|---|---|---|---|---|---|---|---|
| Shahbazi et al. [40] | 2021 | Iran | 2020/6-2021/1 | 109 | NR | Male (53%) | NR | RT-PCR | NA | Malnutrition (Y/N) | GLIM | 61.50% | 61.5% |
| Gómez-Uranga et al. [41] | 2022 | Spain | 2020/4/2-2021/2/28 | 101 | ≤60 years:37.6%;61–74 years:31.7%;≥75 years:30.7% | Male (67.3%) | NR | PCR | NA | Malnutrition (Y/N) | GLIM | 49.5% | 49.5% |
| Czapla et al. [42] | 2021 | Poland | 2020/9-2021/6 | 286 | NR | Male (67.8%) | NR | RT-PCR | NA | Malnutrition risk(Y/N) | NRS-2002; ⩾3 = at risk of malnutrition | 90.2% | 90.2% |
| Martins et al. [43] | 2022 | Brazil | 2020/3-2020/10 | 73 | 56 | Male (63%) | NR | PCR | NA | Malnutrition risk(Y/N) | NRS-2002;<3 = no malnutrition risk, ≥3 = at risk of malnutrition | 85.0% | 85.0% |
| Mohammadi et al. [44] | 2022 | Iran | 2021/4-2021/9 | 110 | 52 ± 15 | Male (59.1%) | NR | NR | NA | Malnutrition risk (Y/N) | NRS-2002; ⩾3 = at risk of malnutrition | 67.3% | 67.3% |
| Martinuzzi et al. [45] | 2021 | Argentine | 2020/3-2020/10 | 285 | 61.24 ± 14.6 | Male (67%) | NR | PCR | NA | Malnutrition (Y/N) | SGA; Grade A =no malnutrition, Grade B= mild/moderate malnutrition,Grade C =severe malnutrition; Malnutrition= SGA score of | 36.9%(35.8%mild/moderate; 1.2%severe) | 36.9% |

| | | | | | | | | | | | | | |
|---|---|---|---|---|---|---|---|---|---|---|---|---|---|
| | | | | | | | | | | | B(mild/moderate) or C (severe) | | |
| **ICU - during hospitalization** | | | | | | | | | | | | | |
| Vahdat et al. [46] | 2022 | Iran | 2021/1-2021/8 | 327 | NR | NR | NR | RT-PCR | NA | Malnutrition risk (Y/N) | GLIM | 59.0% | 59.0% |
| Chadli et al. [47] | 2021 | Morocco | 2020/4/17-2020/5/26 | 41 | 55(19-85) | Male (51.2 %) | NR | NR | 7.3%Mild; 41.5%Moderate; 39%Severe; 12.2%Critical | Malnutrition(no/risk/yes) | MNA;24 - 30 = no malnutrition, 17 - 23.5= at risk of malnutrition, < 16= malnutrition | 19.5% no malnutrition;65.9% malnutrition risk; 14.6% malnutrition | 80.5% |
| Leoni et al. [48] | 2022 | Italy | 2020/3/1-2020/5/31 | 98 | 66 (56–73) | Male (82.7 %) | NR | RT-PCR | NA | Malnutrition risk (low/high) | mNUTRIC;≥5 =high risk , <5 =low risk | 41.8% high risk, 58.2% low risk | 41.8% |
| Zhang et al. [49] | 2021 | China | 2020/1/28-2020/2/21 | 136 | 69 (57–77) | Male (63%) | NR | Guidance for Coronavirus Disease 2019 (6th edition) | NA | Malnutrition risk (low/high) | mNUTRIC;≥5 =high risk , <5 =low risk | 61% high risk, 39% low risk | 61.0% |
| Li et al. [50] | 2021 | China | NR | 211 | NR | Male (56.4 %) | NR | NR | NA | Malnutrition risk (low/high) | mNUTRIC;0-4 =low risk, 5-9 =high risk | 61.6% low risk, 38.9% high risk | 38.9% |
| Cuerda et al. [51] | 2021 | Spain | 2020/3/1-2020/6/30 | 176 | 60.3 ± 10.5 | Male (71.6 %) | NR | NR | NA | Malnutrition risk (low/medium/high) | MUST | 6.8% low risk;9.7%medium risk;83.5% high risk | 93.2% |
| Wu et al. [52] | 2021 | China | 2020/1/15-2020/2/29 | 27 | 74.9±10.5 | Male (66.7 %) | NR | NR | NA | Malnutrition risk (potential/high) | NRS- 2002; ≥3 = potential nutrition risk, ≥5 = high nutrition risk | 3.7% potential risk; 96.3% high risk | 96.3% |

| | | | | | | | | | | | | | |
|---|---|---|---|---|---|---|---|---|---|---|---|---|---|
| Shabanpur et al. [35] | 2022 | Iran | 2021/5-2021/7 | 70 | NR | NR | NR | NR | NA | Malnutrition (risk/moderate/severe) | NRS-2002;<3 = at risk, 3–4 = moderate malnutrition, ≥ 5 = severe malnutrition | 0% at risk; 20% moderate malnutrition; 80% severe malnutrition | 100.0% |
| Pironi et al. [34] | 2021 | Italy | 2020/4 | 106 | NR | NR | NR | NR | NA | Malnutrition risk(Y/N) | NRS-2002;<3 = no malnutrition risk, ≥3 = at risk of malnutrition | 92.5% | 92.5% |
| Alikiaii et al. [53] | 2021 | Iran | 2021/1/1 | 73 | 58.9±18.8 | Male (63%) | NR | RT-PCR | 67.1% Moderate; 32.9% Severe | Malnutrition risk (low/moderate/high) | NRS-2002; ≥3 = malnutrition or at risk of malnutrition | 17.8% low risk; 69.9% moderate risk; 12.3% high risk | 82.2% |

[1] Data are presented as mean ± SD or median (IQR) unless otherwise specified.

[2] Unified prevalence is the proportion at moderate or high risk by various assessment tools.

COVID-19, coronavirus disease 2019;WHO, World Health Organization; ICU, intensive care unit; RT-PCR, reverse transcription polymerase chain reaction; PCR, polymerase chain reaction; CT, computed tomography; NR, not reported; NA, not applicable; BIVA, Bioelectrical Impedance Vector Analysis; MI, body mass index ;CONUT, controlling nutritional status score; GLIM, Global Leadership Initiative on Malnutrition; GNRI, Geriatric Nutritional Risk Index; MNA, Mini Nutritional Assessment; MNA-SF, MNA-short form; mNUTRIC, Modified Nutrition Risk in the Critically ill; MUST, Malnutrition Universal Screening Tool; NCPT, nutrition care process terminology;NRS-2002, Nutritional Risk Score - 2002; PNI, prognostic nutritional index; SGA, Subjective Global Assessment.

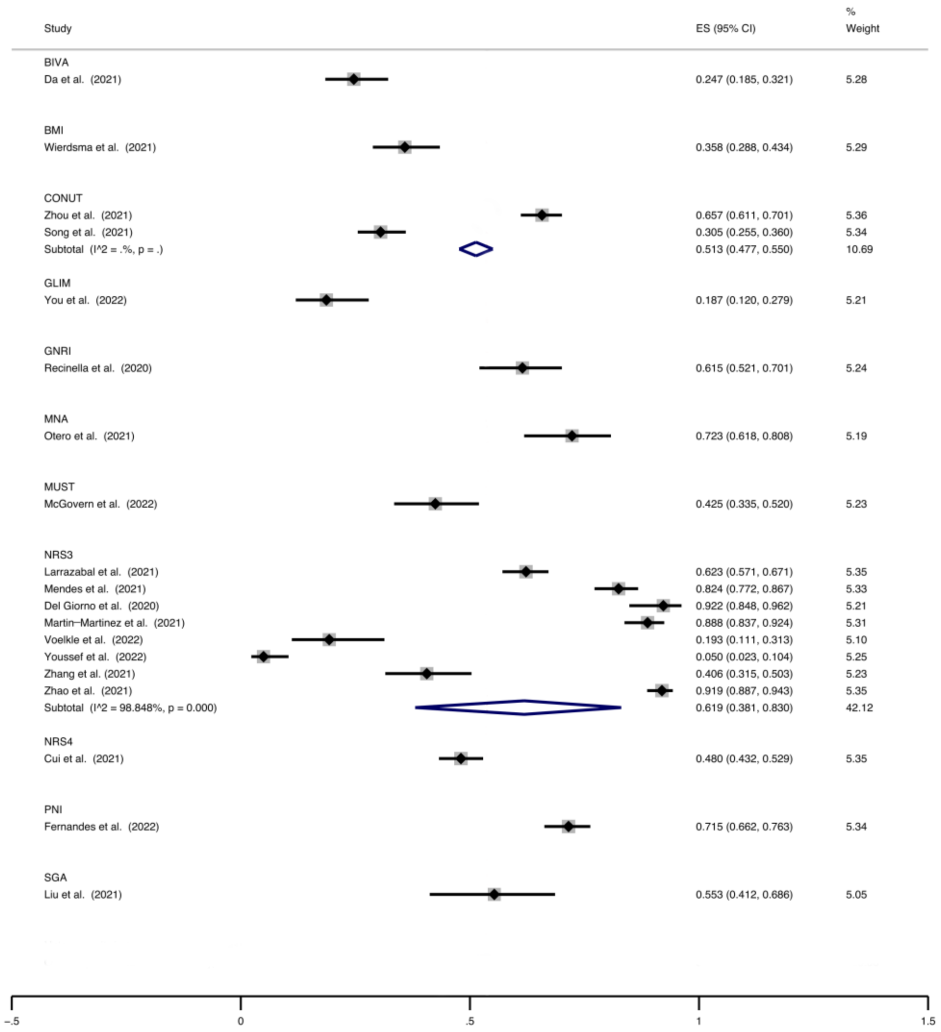


**Figure S1. Meta-Analysis of the Prevalence of Malnutrition or Risk of Malnutrition in COVID-19 Patients (General ward - at admission) by different assessment tools.** BIVA, Bioelectrical Impedance Vector Analysis; BMI, body mass index; CONUT, controlling nutritional status score; GLIM, Global Leadership Initiative on Malnutrition; GNRI, Geriatric Nutritional Risk Index; MNA, Mini Nutritional Assessment; MUST, Malnutrition Universal Screening Tool; NRS3, Nutritional Risk Score – 2002, ≥3 = at risk of malnutrition; NRS4, Nutritional Risk Score – 2002, ≥4 = at risk of malnutrition; PNI, prognostic nutritional index; SGA, Subjective Global Assessment.

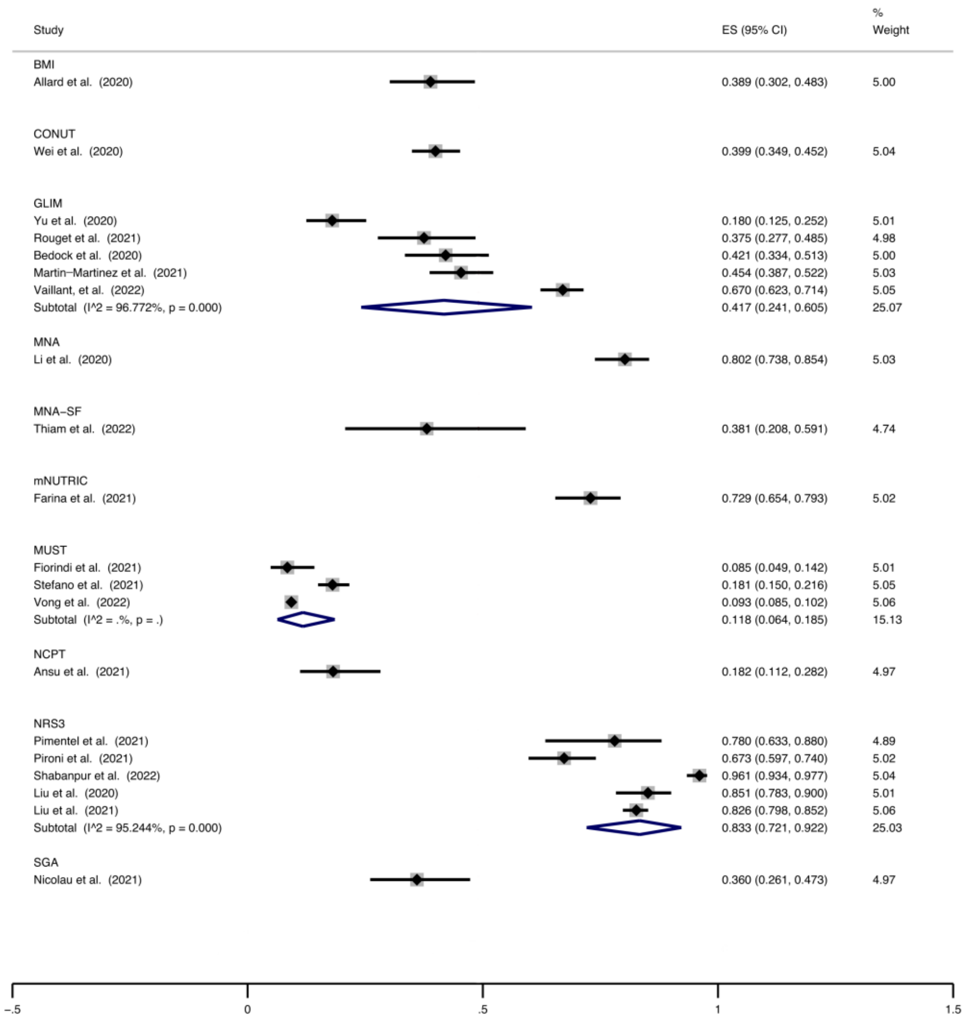


**Figure S2. Meta-Analysis of the Prevalence of Malnutrition or Risk of Malnutrition in COVID-19 Patients (General ward - during hospitalization) by different assessment tools.** BMI, body mass index; CONUT, controlling nutritional status score; GLIM, Global Leadership Initiative on Malnutrition; MNA, Mini Nutritional Assessment; MNA-SF, MNA-short form; mNUTRIC, Modified Nutrition Risk in the Critically ill; MUST, Malnutrition Universal Screening Tool; NCPT, nutrition care process terminology; NRS3, Nutritional Risk Score – 2002, ≥3 = at risk of malnutrition; SGA, Subjective Global Assessment.

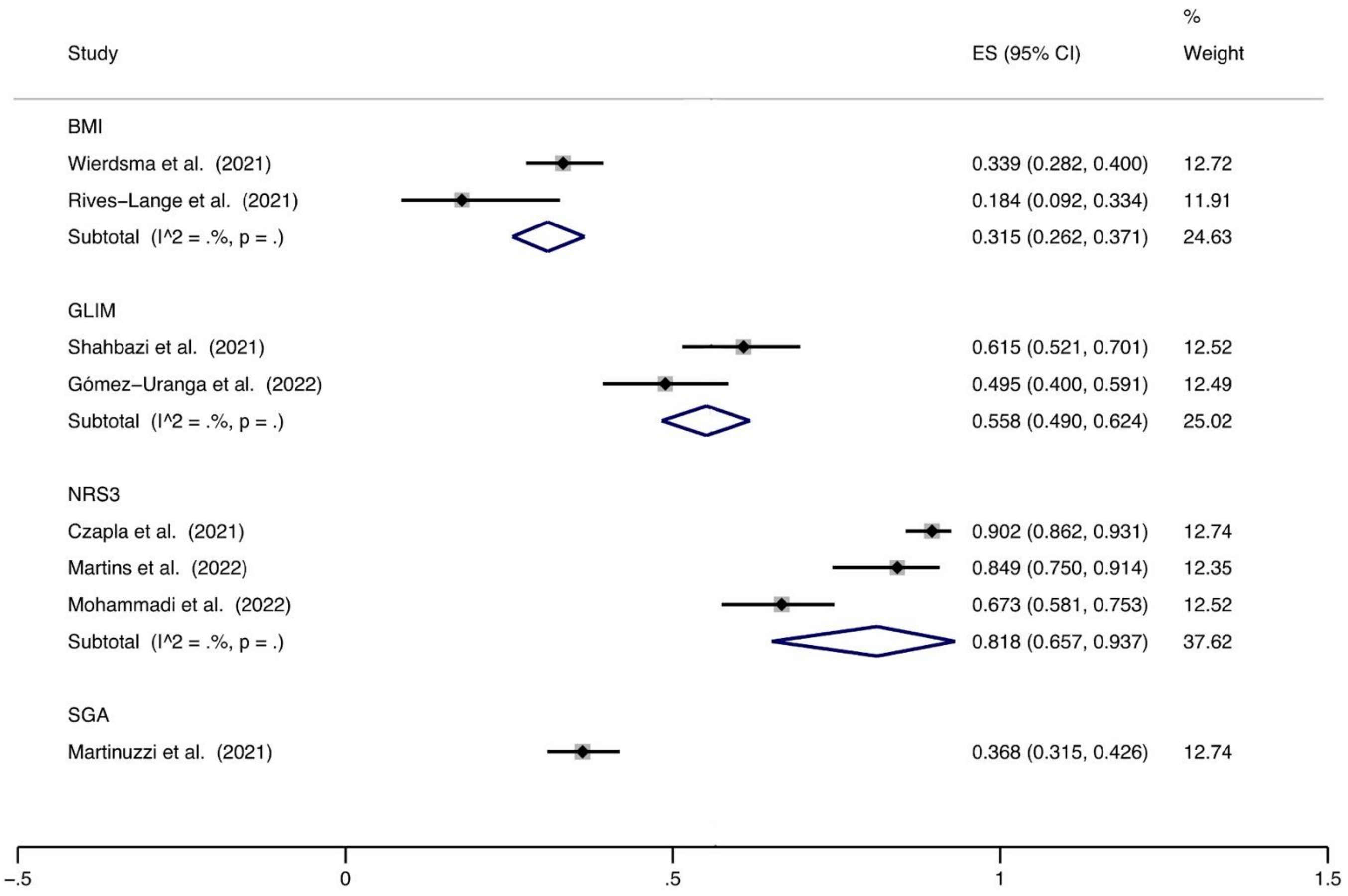


**Figure S3. Meta-Analysis of the Prevalence of Malnutrition or Risk of Malnutrition in COVID-19 Patients (ICU – at admission) by different assessment tools.** BMI, body mass index; GLIM, Global Leadership Initiative on Malnutrition; NRS3, Nutritional Risk Score – 2002, ≥3 = at risk of malnutrition; SGA, Subjective Global Assessment.

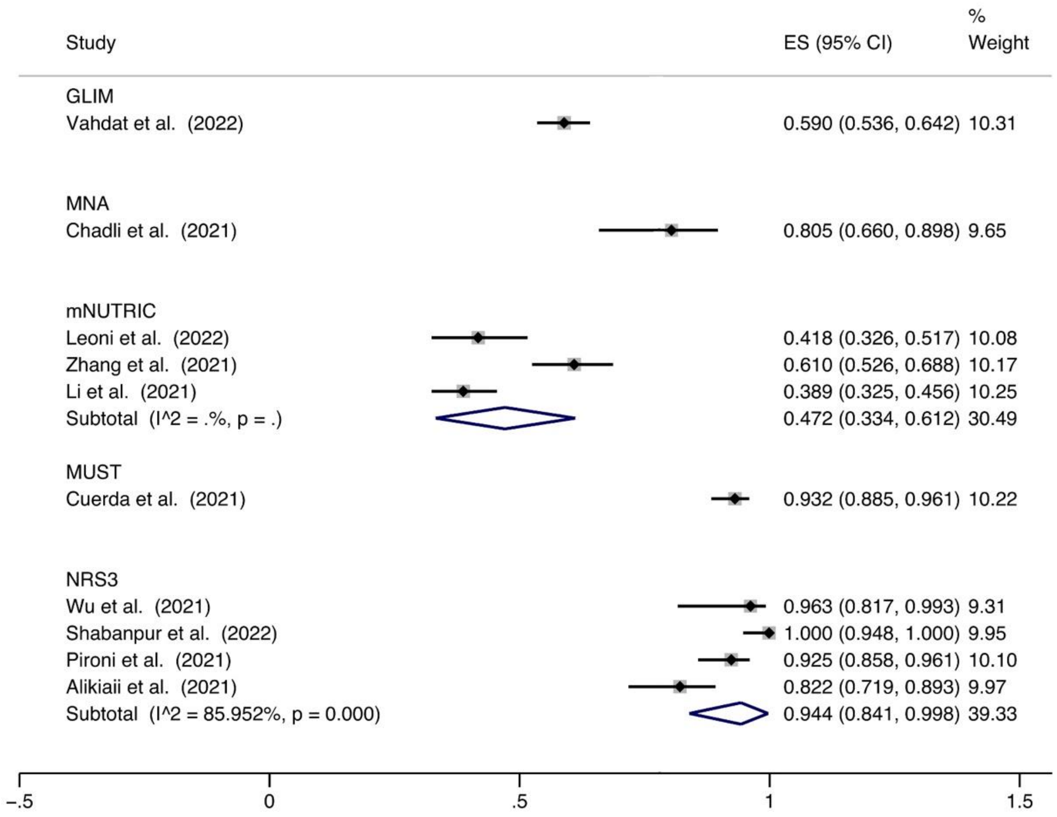


**Figure S4. Meta-Analysis of the Prevalence of Malnutrition or Risk of Malnutrition in COVID-19 Patients (ICU - during hospitalization) by different assessment tools.** GLIM, Global Leadership Initiative on Malnutrition; MNA, Mini Nutritional Assessment; mNUTRIC, Modified Nutrition Risk in the Critically ill; MUST, Malnutrition Universal Screening Tool; NRS3, Nutritional Risk Score – 2002, ≥3 = at risk of malnutrition.

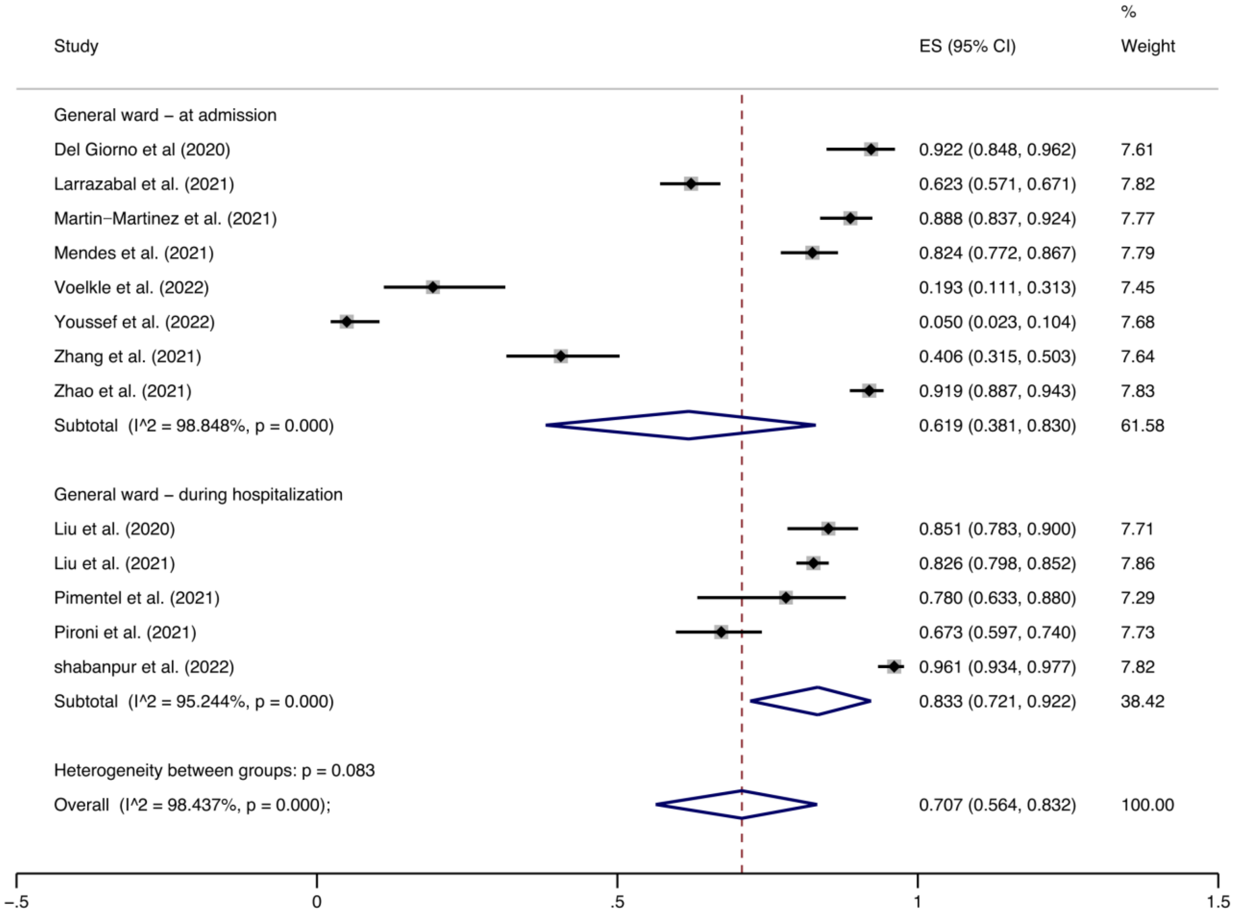


**Figure S5. Meta-Analysis of the Prevalence of Malnutrition or Risk of Malnutrition in COVID-19 Patients (General ward - at admission vs. General ward - during hospitalization) by Nutritional Risk Score – 2002.**

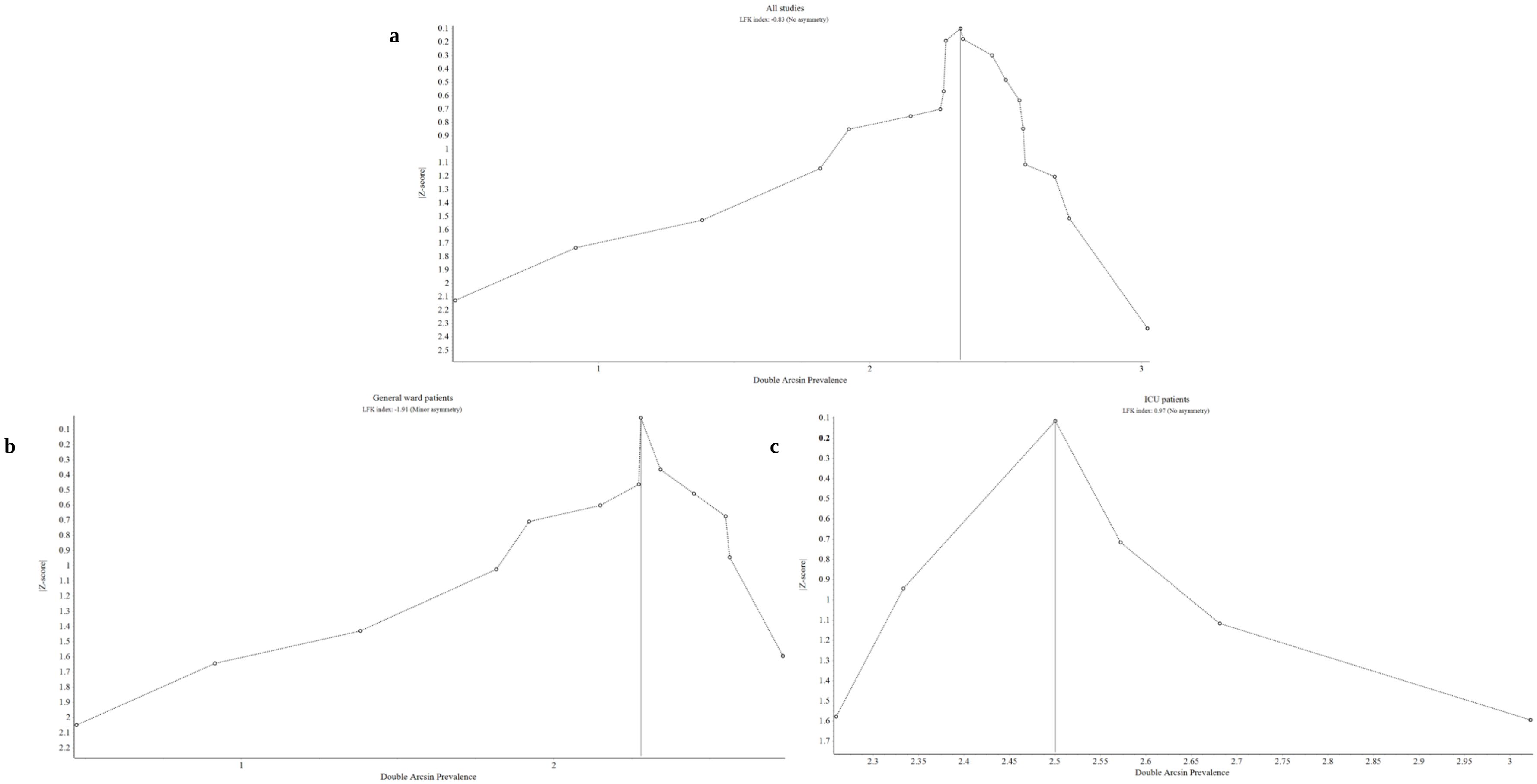


**Figure S6. Doi plot assessing publication bias of included studies.** (**a**) publication bias of all included studies; (**b**) publication bias of general ward studies; (**c**) publication bias of ICU studies. ICU, intensive care unit; LFK index, Luis Furuya-Kanamori index.

## Reference


1. Da Porto, A.; Tascini, C.; Peghin, M.; Sozio, E.; Colussi, G.; Casarsa, V.; Bulfone, L.; Graziano, E.; De Carlo, C.; Catena, C., et al. Prognostic Role of Malnutrition Diagnosed by Bioelectrical Impedance Vector Analysis in Older Adults Hospitalized with COVID-19 Pneumonia: A Prospective Study. *Nutrients* **2021**, *13*, doi:10.3390/nu13114085.
2. Wierdsma, N.J.; Kruizenga, H.M.; Konings, L.A.; Krebbers, D.; Jorissen, J.R.; Joosten, M.I.; van Aken, L.H.; Tan, F.M.; van Bodegraven, A.A.; Soeters, M.R., et al. Poor nutritional status, risk of sarcopenia and nutrition related complaints are prevalent in COVID-19 patients during and after hospital admission. *Clin Nutr ESPEN* **2021**, *43*, 369-376, doi:10.1016/j.clnesp.2021.03.021.
3. Zhou, J.; Ma, Y.; Liu, Y.; Xiang, Y.; Tao, C.; Yu, H.; Huang, J. A Correlation Analysis between the Nutritional Status and Prognosis of COVID-19 Patients. *J Nutr Health Aging* **2021**, *25*, 84-93, doi:10.1007/s12603-020-1457-6.
4. Song, F.; Ma, H.; Wang, S.; Qin, T.; Xu, Q.; Yuan, H.; Li, F.; Wang, Z.; Liao, Y.; Tan, X., et al. Nutritional screening based on objective indices at admission predicts in-hospital mortality in patients with COVID-19. *Nutr J* **2021**, *20*, 46.
5. You, Y.; Chen, M.; Chen, X.; Yu, W. Diaphragm thickness on computed tomography for nutritional assessment and hospital stay prediction in critical COVID-19. *Asia Pac J Clin Nutr* **2022**, *31*, 33-40, doi:10.6133/apjcn.202203_31(1).0004.
6. Recinella, G.; Marasco, G.; Serafini, G.; Maestri, L.; Bianchi, G.; Forti, P.; Zoli, M. Prognostic role of nutritional status in elderly patients hospitalized for COVID-19: a monocentric study. *Aging Clin Exp Res* **2020**, *32*, 2695-2701, doi:10.1007/s40520-020-01727-5.
7. Otero, J.A.; Figuero, L.S.B.; Mattin, M.G.; Martin, I.U.; Morais, P.C.; Olmedo, L.C.; Galiana, L.I.; Gutierrez, C.D.; Gomez, J.C.; Gudino, L.C., et al. The nutritional status of the elderly patient infected with COVID-19: the forgotten risk factor? *Figshare* **2021**, 10.6084/m9.figshare.13663538.v2, doi:10.6084/m9.figshare.13663538.v2.
8. McGovern, J.; Al-Azzawi, Y.; Kemp, O.; Moffitt, P.; Richards, C.; Dolan, R.D.; Laird, B.J.; McMillan, D.C.; Maguire, D. The relationship between frailty, nutritional status, co-morbidity, CT-body composition and systemic inflammation in patients with COVID-19. *J Transl Med* **2022**, *20*, 98, doi:10.1186/s12967-022-03300-2.
9. Larrazabal, R.B., Jr.; Chiu, H.H.C.; Palileo-Villanueva, L.A.M. Outcomes of nutritionally at-risk Coronavirus Disease 2019 (COVID 19) patients admitted in a tertiary government hospital: A follow-up study of the MalnutriCoV study. *Clin Nutr ESPEN* **2021**, *43*, 239-244, doi:10.1016/j.clnesp.2021.04.008.
10. Mendes, A.; Serratrice, C.; Herrmann, F.R.; Gold, G.; Graf, C.E.; Zekry, D.; Genton, L. Nutritional risk at hospital admission is associated with prolonged length of hospital stay in old patients with COVID-19. *Clin Nutr* **2021**, 10.1016/j.clnu.2021.03.017, doi:10.1016/j.clnu.2021.03.017.
11. Del Giorno, R.; Quarenghi, M.; Stefanelli, K.; Capelli, S.; Giagulli, A.; Quarleri, L.; Stehrenberger, D.; Ossola, N.; Monotti, R.; Gabutti, L. Nutritional Risk Screening and Body Composition in COVID-19 Patients Hospitalized in an Internal Medicine Ward. *Int J Gen Med* **2020**, *13*, 1643-1651, doi:10.2147/ijgm.S286484.
12. Martin–Martinez, A.; Ortega, O.; Viñas, P.; Arreola, V.; Nascimento, W.; Costa, A.; Riera, S.A.; Alarcón, C.; Clavé, P. COVID-19 is associated with oropharyngeal dysphagia and malnutrition in hospitalized patients during the spring 2020 wave of the pandemic. *Clinical Nutrition* **2021**, 10.1016/j.clnu.2021.06.010, doi:10.1016/j.clnu.2021.06.010.
13. Voelkle, M.; Gregoriano, C.; Neyer, P.; Koch, D.; Kutz, A.; Bernasconi, L.; Conen, A.; Mueller, B.; Schuetz, P. Prevalence of Micronutrient Deficiencies in Patients Hospitalized with COVID-19: An Observational Cohort Study. *Nutrients* **2022**, *14*, doi:10.3390/nu14091862.
14. Youssef, N.; Elbadry, M.; Al Shafie, A.; Abdalazeem, A.; Hasan, S.; Tahoon, M.; Omran, D.; El Kassas, M. Nutritional status associated with clinical outcomes among patients hospitalized with COVID-19: A multicenter prospective study in Egypt. *Nurs Health Sci* **2022**, *24*, 204-213, doi:10.1111/nhs.12913.
15. Zhang, K.; Qin, W.; Zheng, Y.; Pang, J.; Zhong, N.; Fei, J.; Li, Y.; Jian, X.; Hou, X.; Hu, Z., et al. Malnutrition Contributes to Low Lymphocyte Count in Early-Stage Coronavirus Disease-2019. *Front Nutr* **2021**, *8*, 739216, doi:10.3389/fnut.2021.739216.

16. Zhao, X.; Li, Y.; Ge, Y.; Shi, Y.; Lv, P.; Zhang, J.; Fu, G.; Zhou, Y.; Jiang, K.; Lin, N., et al. Evaluation of Nutrition Risk and Its Association With Mortality Risk in Severely and Critically Ill COVID-19 Patients. *JPEN J Parenter Enteral Nutr* **2021**, *45*, 32-42, doi:10.1002/jpen.1953.
17. Cui, N.; Tong, H.; Li, Y.; Ge, Y.; Shi, Y.; Lv, P.; Zhao, X.; Zhang, J.; Fu, G.; Zhou, Y., et al. Role of Prealbumin in Predicting the Prognosis of Severely and Critically Ill COVID-19 Patients. *Am J Trop Med Hyg* **2021**, *105*, 718-726, doi:10.4269/ajtmh.21-0234.
18. Fernandes, A.L.; Reis, B.Z.; Murai, I.H.; Pereira, R.M.R. Prognostic Nutritional Index and Oxygen Therapy Requirement Associated With Longer Hospital Length of Stay in Patients With Moderate to Severe COVID-19: Multicenter Prospective Cohort Analyses. *Front Nutr* **2022**, *9*, 802562, doi:10.3389/fnut.2022.802562.
19. Liu, H.; Zhou, L.; Wang, H.; Wang, X.; Qu, G.; Cai, J.; Zhang, H. Malnutrition is associated with hyperinflammation and immunosuppression in COVID-19 patients: A prospective observational study. *Nutr Clin Pract* **2021**, *36*, 863-871, doi:10.1002/ncp.10679.
20. Allard, L.; Ouedraogo, E.; Molleville, J.; Bihan, H.; Giroux-Leprieur, B.; Sutton, A.; Baudry, C.; Josse, C.; Didier, M.; Deutsch, D., et al. Malnutrition: Percentage and Association with Prognosis in Patients Hospitalized for Coronavirus Disease 2019. *Nutrients* **2020**, *12*, doi:10.3390/nu12123679.
21. Wei, C.; Liu, Y.; Li, Y.; Zhang, Y.; Zhong, M.; Meng, X. Evaluation of the nutritional status in patients with COVID-19. *J Clin Biochem Nutr* **2020**, *67*, 116-121, doi:10.3164/jcbn.20-91.
22. Yu, Y.; Ye, J.; Chen, M.; Jiang, C.; Lin, W.; Lu, Y.; Ye, H.; Li, Y.; Wang, Y.; Liao, Q., et al. Malnutrition Prolongs the Hospitalization of Patients with COVID-19 Infection: A Clinical Epidemiological Analysis. *Journal of Nutrition, Health and Aging* **2020**, 10.1007/s12603-020-1541-y, doi:10.1007/s12603-020-1541-y.
23. Rouget, A.; Vardon-Bounes, F.; Lorber, P.; Vavasseur, A.; Marion, O.; Marcheix, B.; Lairez, O.; Balardy, L.; Fourcade, O.; Conil, J.-M. Prevalence of malnutrition in coronavirus disease 19: The NUTRICOV study. *British Journal of Nutrition* **2021**, *126*, 1296-1303.
24. Bedock, D.; Bel Lassen, P.; Mathian, A.; Moreau, P.; Couffignal, J.; Ciangura, C.; Poitou-Bernert, C.; Jeannin, A.C.; Mosbah, H.; Fadlallah, J., et al. Prevalence and severity of malnutrition in hospitalized COVID-19 patients. *Clin Nutr ESPEN* **2020**, *40*, 214-219, doi:10.1016/j.clnesp.2020.09.018.
25. Vaillant, M.-F.; Agier, L.; Martineau, C.; Philipponneau, M.; Romand, D.; Masdoua, V.; Behar, M.; Nesseler, C.; Achamrah, N.; Laubé, V. Food intake and weight loss of surviving inpatients in the course of COVID-19 infection: A longitudinal study of the multicenter NutriCoviD30 cohort. *Nutrition* **2022**, *93*, 111433.
26. Li, T.; Zhang, Y.; Gong, C.; Wang, J.; Liu, B.; Shi, L.; Duan, J. Prevalence of malnutrition and analysis of related factors in elderly patients with COVID-19 in Wuhan, China. *Eur J Clin Nutr* **2020**, *74*, 871-875, doi:10.1038/s41430-020-0642-3.
27. Thiam, C.N.; Mathavan, S.; Abdullah, A.; Chong, E.G.M. Malnutrition among patients admitted to the subacute geriatric ward during the COVID-19 pandemic era: A cross-sectional study in a tertiary hospital in Malaysia. *The Medical journal of Malaysia* **2022**, *77*, 313-319.
28. Farina, N.; Nordbeck, S.; Montgomery, M.; Cordwin, L.; Blair, F.; Cherry-Bukowiec, J.; Kraft, M.D.; Pleva, M.R.; Raymond, E. Early Enteral Nutrition in Mechanically Ventilated Patients With COVID-19 Infection. *Nutrition in Clinical Practice* **2021**, *36*, 440-448, doi:10.1002/ncp.10629.
29. Fiorindi, C.; Campani, F.; Rasero, L.; Campani, C.; Livi, L.; Giovannoni, L.; Amato, C.; Giudici, F.; Bartoloni, A.; Fattirolli, F., et al. Prevalence of nutritional risk and malnutrition during and after hospitalization for COVID-19 infection: Preliminary results of a single-centre experience. *Clin Nutr ESPEN* **2021**, *45*, 351-355, doi:10.1016/j.clnesp.2021.07.020.
30. Stefano, M.; Andrea, B.; Daniela, C.; Emanuela, M.; Lorena, P.; Daniela, D.; Sökeland, F.; Azzolini, E.; Beatrice, M. Malnutrition risk as a negative prognostic factor in COVID-19 patients. *Clin Nutr ESPEN* **2021**, *45*, 369-373, doi:10.1016/j.clnesp.2021.07.016.
31. Vong, T.; Yanek, L.R.; Wang, L.; Yu, H.; Fan, C.; Zhou, E.; Oh, S.J.; Szvarca, D.; Kim, A.; Potter, J.J., et al. Malnutrition Increases Hospital Length of Stay and Mortality among Adult Inpatients with COVID-19. *Nutrients* **2022**, *14*, doi:10.3390/nu14061310.
32. Ansu, V.; Papoutsakis, C.; Gletsu-Miller, N.; Spence, L.A.; Kelley, K.; Woodcock, L.; Wallace, T.C.; Steiber, A. Nutrition care practice patterns for patients with COVID-19—A

preliminary report. *Journal of Parenteral and Enteral Nutrition* **2021**, *45*, 1774-1778, doi:10.1002/jpen.2106.

33. Pimentel, R.F.W.; Moraes, G.C.D.; Barcelos, S.G.C.; Figueiredo, P.C.M.D.; Das Merces, M.C. Evaluation of Nutritional Risk and Prevalence of Obesity in Patients with Covid-19 in A Reference Hospital in Salvador, Bahia, Brazil: A Cross-Sectional Study. *International Journal of Nutrology* **2021**, *14*, 11-15, doi:10.1055/s-0041-1728680.
34. Pironi, L.; Sasdelli, A.S.; Ravaioli, F.; Baracco, B.; Battaiola, C.; Bocedi, G.; Brodosi, L.; Leoni, L.; Mari, G.A.; Musio, A. Malnutrition and nutritional therapy in patients with SARS-CoV-2 disease. *Clin Nutr* **2021**, *40*, 1330-1337, doi:10.1016/j.clnu.2020.08.021.
35. shabanpur, M.; Pourmahmoudi, A.; Nicolau, J.; Veronese, N.; Roustaei, N.; Jahromi, A.J.; Hosseinikia, M. The importance of nutritional status on clinical outcomes among both ICU and Non-ICU patients with COVID-19. *Clinical Nutrition ESPEN* **2022**, *49*, 225-231, doi:10.1016/j.clnesp.2022.04.016.
36. Liu, G.; Zhang, S.; Mao, Z.; Wang, W.; Hu, H. Clinical significance of nutritional risk screening for older adult patients with COVID-19. *Eur J Clin Nutr* **2020**, *74*, 876-883, doi:10.1038/s41430-020-0659-7.
37. Liu, A.; Cong, J.; Wang, Q.; Mei, Y.; Peng, Y.; Zhou, M.; Zhu, W.; Chen, X.; Guan, W.; He, P. Risk of Malnutrition Is Common in Patients with Coronavirus Disease 2019 (COVID-19) in Wuhan, China: A Cross-sectional Study. *Journal of Nutrition* **2021**, *151*, 1591-1596, doi:10.1093/jn/nxab009.
38. Nicolau, J.; Ayala, L.; Sanchis, P.; Olivares, J.; Dotres, K.; Soler, A.-G.; Rodriguez, I.; Gomez, L.-A.; Masmiquel, L. Influence of nutritional status on clinical outcomes among hospitalized patients with COVID-19. *Clinical Nutrition Espen* **2021**, *43*, 223-229, doi:10.1016/j.clnesp.2021.04.013.
39. Rives-Lange, C.; Zimmer, A.; Merazka, A.; Carette, C.; Martins-Bexinga, A.; Hauw-Berlemont, C.; Guerot, E.; Jannot, A.S.; Diehl, J.L.; Czernichow, S., et al. Evolution of the nutritional status of COVID-19 critically-ill patients: A prospective observational study from ICU admission to three months after ICU discharge. *Clin Nutr* **2021**, 10.1016/j.clnu.2021.05.007, doi:10.1016/j.clnu.2021.05.007.
40. Shahbazi, S.; Hajimohammadebrahim-Ketabforoush, M.; Shariatpanahi, M.V.; Shahbazi, E.; Shariatpanahi, Z.V. The validity of the global leadership initiative on malnutrition criteria for diagnosing malnutrition in critically ill patients with COVID-19: A prospective cohort study. *Clinical Nutrition Espen* **2021**, *43*, 377-382, doi:10.1016/j.clnesp.2021.03.020.
41. Gómez-Uranga, A.; Guzmán-Martínez, J.; Esteve-Atiénzar, P.J.; Wikman-Jorgensen, P.; Núñez-Cruz, J.M.; Espinosa-Del-Barrio, L.; Hernández-Isasi, I.; Pomares-Gómez, F.J.; Perelló-Camacho, E.; Fernández-García, N., et al. Nutritional and Functional Impact of Acute SARS-CoV-2 Infection in Hospitalized Patients. *J Clin Med* **2022**, *11*, doi:10.3390/jcm11092424.
42. Czapla, M.; Juárez-Vela, R.; Gea-Caballero, V.; Zieliński, S.; Zielińska, M. The Association between Nutritional Status and In-Hospital Mortality of COVID-19 in Critically-Ill Patients in the ICU. *Nutrients* **2021**, *13*, doi:10.3390/nu13103302.
43. Martins, P.M.; Gomes, T.L.N.; Franco, E.P.; Vieira, L.L.; Pimentel, G.D. High neutrophil-to-lymphocyte ratio at intensive care unit admission is associated with nutrition risk in patients with COVID-19. *Journal of Parenteral and Enteral Nutrition* **2022**, 10.1002/jpen.2318, doi:10.1002/jpen.2318.
44. Mohammadi, P.; Varpaei, H.A.; Mohammadi, M.; Rahimi, M.; Orandi, A. Evaluation of the Relationship between Nutritional Status of COVID-19 Patients Admitted to the ICU and Patients' Prognosis: A Cohort Study. *Journal of Nutrition and Metabolism* **2022**, *2022*.
45. Martinuzzi, A.L.N.; Manzanares, W.; Quesada, E.; Reberendo, M.J.; Baccaro, F.; Aversa, I.; Kecskes, C.E.; Magnífico, L.; González, V.; Bolzico, D., et al. Nutritional risk and clinical outcomes in critically ill adult patients with COVID-19. *Nutr Hosp* **2021**, *38*, 1119-1125, doi:10.20960/nh.03749.
46. Vahdat Shariatpanahi, Z.; Vahdat Shariatpanahi, M.; Shahbazi, E.; Shahbazi, S. Refeeding Syndrome and Its Related Factors in Critically Ill Coronavirus Disease 2019 Patients: A Prospective Cohort Study. *Front Nutr* **2022**, *9*, 830457, doi:10.3389/fnut.2022.830457.
47. Chadli, A.; Haraj, N.E.; El Aziz, S.; Laidi, S.; Mounir, A.; Bensbaa, S.; Mjabber, A.; Barrou, L.; El Kettani El Hamidi, C.; Nsiri, A., et al. COVID-19: Patient care after discharge from the Intensive Care Unit. *Int J Clin Pract* **2021**, *75*, e14270, doi:10.1111/ijcp.14270.
48. Leoni, M.L.G.; Moschini, E.; Beretta, M.; Zanello, M.; Nolli, M. The modified NUTRIC score (mNUTRIC) is associated with increased 28-day mortality in critically ill COVID-19

patients: Internal validation of a prediction model. *Clin Nutr ESPEN* **2022**, *48*, 202-209, doi:10.1016/j.clnesp.2022.02.014.

49. Zhang, P.; He, Z.; Yu, G.; Peng, D.; Feng, Y.; Ling, J.; Wang, Y.; Li, S.; Bian, Y. The modified NUTRIC score can be used for nutritional risk assessment as well as prognosis prediction in critically ill COVID-19 patients. *Clinical Nutrition* **2021**, *40*, 534-541.
50. Li, G.; Zhou, C.-L.; Ba, Y.-M.; Wang, Y.-M.; Song, B.; Cheng, X.-B.; Dong, Q.-F.; Wang, L.-L.; You, S.-S. Nutritional risk and therapy for severe and critical COVID-19 patients: A multicenter retrospective observational study. *Clinical Nutrition* **2021**, *40*, 2154-2161, doi:10.1016/j.clnu.2020.09.040.
51. Cuerda, C.; Sánchez López, I.; Gil Martínez, C.; Merino Viveros, M.; Velasco, C.; Cevallos Peñafiel, V.; Maíz Jiménez, M.; Gonzalo, I.; González-Sánchez, V.; Ramos Carrasco, A., et al. Impact of COVID-19 in nutritional and functional status of survivors admitted in intensive care units during the first outbreak. Preliminary results of the NUTRICOVID study. *Clin Nutr* **2021**, 10.1016/j.clnu.2021.11.017, doi:10.1016/j.clnu.2021.11.017.
52. Wu, S.; Lou, J.; Xu, P.; Luo, R.; Li, L. Early enteral nutrition improves the outcome of critically ill patients with COVID-19: A retrospective study. *Asia Pac J Clin Nutr* **2021**, *30*, 192-198, doi:10.6133/apjcn.202106_30(2).0002.
53. Alikiaii, B.; Heidari, Z.; Fazeli, A.; Rahimi Varposhti, M.; Moradi Farsani, D.; Fattahpour, S.; Rafiee, S.; Bagherniya, M. Evaluation of the effectiveness of the Nutritional Risk Screening System 2002 (NRS-2002) in COVID-19 patients admitted to the intensive care unit. *Int J Clin Pract* **2021**, *75*, e14934, doi:10.1111/ijcp.14934.
